# Editorial: Periodontitis and cardiovascular disease: shared clinical challenges in patient care

**DOI:** 10.3389/fdmed.2025.1613091

**Published:** 2025-05-20

**Authors:** Gaetano Isola, Alessandro Polizzi

**Affiliations:** Unit of Periodontology, Department of General Surgery and Medical-Surgical Specialities, University of Catania, Catania, Italy

**Keywords:** periodontitis, periodontal treatment, cardiovascular disease, systemic disease, biomarkers

**Editorial on the Research Topic**
Periodontitis and cardiovascular disease: shared clinical challenges in patient care

During the last few decades, increasingly mechanistic investigations have uncovered shared biological pathways that explain how periodontal disease can influence cardiovascular health and vice versa. The intersection of periodontitis and cardiovascular disease (CVD) represents a pressing concern in modern healthcare (1). Both conditions are highly prevalent in adult populations, particularly among those with shared risk factors like diabetes, smoking, and age with high risk also on cancer (2). As evidence mounts regarding the biological connection between these diseases, it is crucial to delve into the mechanisms that underlie this relationship, the impact of periodontal treatment on cardiovascular outcomes (3), and the shared clinical challenges faced by healthcare providers in managing patients with both conditions.

Periodontitis is a chronic, multifactorial disease of the periodontal tissues triggered by a specific host response (4, 5) following the presence of pathogenic bacteria (6). These bacteria can invade the bloodstream through bleeding gums, leading to a systemic inflammatory response. Inflammatory markers like C-reactive protein (CRP) and interleukins are elevated in both periodontitis and CVD (7), further amplifying the systemic inflammatory burden (8, 9). This inflammation plays a critical role in the development and progression of atherosclerosis—the buildup of fatty plaques in the arteries—which can lead to heart attacks and strokes. A key mechanism that links periodontitis to CVD is the impact of oral bacteria on endothelial function (5, 10). Endothelial cells line the blood vessels and play a critical role in maintaining vascular health. The introduction of bacterial endotoxins from periodontal infections can damage endothelial cells, contributing to atherosclerotic plaque formation with certain immunomodulatory pathways (11, 12). Furthermore, these bacterial components can induce the production of inflammatory cytokines, leading to further vascular damage and enhancing the risk of clot formation. Thus, the biological pathway that connects periodontitis and peri-implant diseases with dysbiosis and related systemic diseases is largely rooted in the inflammatory and microbial interplay. In this regard, periodontitis has been associated with a plethora of systemic diseases (13) such as CVD, diabetes, bowel disease (14) and, at the same time, patients with CVD often have comorbidities, such as diabetes or hypertension, which exacerbate periodontal disease.

This Research Topic was aimed at widening the knowledge of the studies examining the association between periodontitis and different manifestations of cardiovascular disease. The issue currently includes 6 articles on the epidemiological studies examining the association between periodontitis and different manifestations of cardiovascular disease and on the mechanistic investigations exploring the biological pathways linking periodontitis and cardiovascular disease. All contributions to this research topic focus on one or more of the research areas highlighted above, as evidenced below by reference to the designated areas. The dual burden of periodontitis and cardiovascular disease presents unique challenges in clinical management. Both conditions require long-term care strategies and multidisciplinary collaboration. Cardiologists, periodontists, and primary care providers must work together to optimize treatment plans for patients, taking into account the potential interactions between diet, medications used for cardiovascular management and those required for periodontal care and possible regeneration (15).

All published studies evaluated how a key challenge was periodontal treatment in CVD patients. Many individuals with periodontitis may not be aware of its link to cardiovascular health, and likewise, those with heart disease may not recognize the importance of oral hygiene in managing their cardiovascular risk. Clinicians must invest in educating patients about the bidirectional relationship between oral health and heart health, emphasizing the importance of both preventive dental care and cardiovascular disease management. Periodontal treatment has been shown to improve endothelial function and reduce levels of inflammatory biomarkers associated with cardiovascular disease. The improvement in endothelial function is of particular importance as it is an early indicator of cardiovascular risk. These findings suggest that managing oral health in patients with cardiovascular disease may help mitigate some of the vascular dysfunction associated with CVD.

The potential benefit of periodontal treatment in improving cardiovascular health outcomes is an area of active research. While a direct causal relationship between periodontal treatment and reduced cardiovascular events is still under investigation, several studies suggest that treating periodontitis can positively impact cardiovascular risk factors.

However, the clinical evidence linking periodontal treatment directly to improved cardiovascular outcomes remains mixed. While there is a clear reduction in inflammatory markers, whether this leads to a significant decrease in the incidence of cardiovascular events is still debated. More robust longitudinal studies are needed to conclusively determine the long-term cardiovascular benefits of periodontal therapy. To better evaluate the impact of periodontal treatment on cardiovascular health outcomes, future research must focus on large-scale, well-designed randomized controlled trials that examine the long-term effects of periodontal therapy on cardiovascular events such as heart attacks and strokes. These studies should include diverse patient populations with both periodontitis and established cardiovascular disease, accounting for variables such as age, comorbidities, and medication use. Moreover, mechanistic studies exploring the pathways through which periodontal treatment may influence systemic inflammation and cardiovascular health are crucial. Investigating how reducing oral bacteria or improving gum health affects the systemic inflammatory response and vascular function could shed light on the specific biological processes that mediate this connection.

In addition, evaluating the economic and clinical outcomes of integrated care models—where periodontists and cardiologists collaborate on the management of patients with both conditions—could provide valuable insights into the effectiveness of such an approach. This could ultimately inform clinical guidelines and help optimize patients' care with periodontitis and cardiovascular disease. In the meantime, a coordinated approach that addresses oral and cardiovascular health may provide the best chance to improve patient outcomes and reduce the burden of these common yet interconnected chronic diseases.
